# Symbiotic bacteria participate in pectinolytic metabolism to enhance larval growth in *Zeugodacus cucurbitae*

**DOI:** 10.1002/ps.70035

**Published:** 2025-07-03

**Authors:** Guangmei Chen, Zhenya Tian, Yang Yue, Xuyuan Gao, Hongsong Chen, Jingfang Yang, Weihua Ma, Dehong Zheng, Huihua Tan, Zhongshi Zhou

**Affiliations:** ^1^ Guangxi Key Laboratory of Agro‐environment and Agro‐Product Safety, College of Agriculture Guangxi University Nanning China; ^2^ National Nanfan Research Institute, Chinese Academy of Agricultural Sciences Sanya China; ^3^ Guangxi Key Laboratory for Biology of Crop Diseases and Insect Pests, Institute of Plant Protection Guangxi Academy of Agricultural Sciences Nanning China; ^4^ State Key Laboratory for Biology of Plant Diseases and Insect Pests, Institute of Plant Protection Chinese Academy of Agricultural Sciences Beijing China; ^5^ College of Plant Science and Technology Huazhong Agricultural University Wuhan China

**Keywords:** *Zeugodacus cucurbitae*, feeding wounds, *Klebsiella*, pectinases, pectinolytic metabolism

## Abstract

**BACKGROUND:**

Symbiotic microbes play a pivotal role in the feeding processes of phytophagous insects, and symbiosis has been established as a key strategy for certain species to acquire pectinases. However, whether symbiotic bacteria play a role in the pectinolytic metabolism of *Zeugodacus cucurbitae* remains unclear.

**RESULTS:**

Removal of symbiotic bacteria *via* egg sterilization significantly reduced larval food consumption, growth, and pectinase activity (*P* < 0.05), highlighting that the microbiota was required for *Z. cucurbitae* larval growth under feeding on host plants. Microbial community analysis identified *Klebsiella spp.* as persistent colonizers of larval feeding wounds, exhibiting recycling between host tissues and plant substrates. Functional assays demonstrated that *Klebsiella* strains (CpL20, CpL49, CpL63, and CpL64) formed distinct hydrolysis zones on pectin medium and degraded pectin *via* high enzymatic activity (495.98–830.54 μ/mL). Reintroduction of *Klebsiella spp.* restored larval growth in sterile treatment groups, confirming their metabolic contribution.

**CONCLUSION:**

Our results suggest that *Klebsiella spp.* circulate between the feeding environment and larval tissues, participating in the pectinolytic metabolism to utilize the host plant efficiently, thereby facilitating larval growth and development. This study provides a foundation for understanding the role of symbiotic bacteria in pectinolytic metabolism during the ecological adaptation of phytophagous insects and offers new insights into the environmentally friendly management of *Z. cucurbitae* in agricultural settings. © 2025 The Author(s). *Pest Management Science* published by John Wiley & Sons Ltd on behalf of Society of Chemical Industry.

## Graphical abstract

The larval food consumption, growth, and pectinase activity without symbiotic bacteria are suppressed in *Zeugodacus cucurbitae*. We isolated four strains of *Klebsiella spp.* that can participate in pectinolytic metabolism from feeding wounds. Reintroducing them to sterile eggs breaks the developmental constraints caused by the lack of symbiotic bacteria. The *Klebsiella spp.* may help the larvae's pectinolytic metabolism to adapt plant diet. These findings provide a theoretical basis for the control strategy of *Z. cucurbitae* based on symbiotic bacteria.

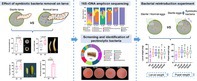

## 1 INTRODUCTION

Insects have fed on plants for over 400 million years. During their transition to phytophagy, insects have needed to overcome numerous challenges associated with feeding on plants, including digesting stubborn plant polymers, providing nutrients that the diet lacks, and degrading plant secondary metabolites.^1^, ^2^, ^3^ To address these challenges, phytophagous insects harbor diverse microbiota in their digestive systems. Symbiotic relationships between these microorganisms and the hosts facilitate their adaptation to plant‐based diets. For example, the gut bacteria of *Cephalotes varians* code a nitrogen‐recycling pathway to recycle urea, and likely uric acid, using recycled nitrogen to synthesize essential amino acids for the host, thereby helping the host to maintain a herbivorous diet.^4^ The gut microbiome of pioneering populations enhances the compatibility of invasive pests, *Hyphantria cunea*, to new hosts and enables more rapid adaptation to new habitats, possibly because these microbiomes are associated with tolerance to plant toxins.^5^ Interestingly, gut bacteria can be transferred to host plants through insect foraging, excretion, or oviposition. Once inside the plants, symbiotic bacteria utilize plant nutrients to transmit throughout the plant, and influence plant‐insect interactions.^6^ A notable example is *Leptinotarsa decemlineata*, which secretes *Pseudomonas sp*. through feeding wounds, thereby manipulating the salicylic acid signaling pathways of tomato (*Solanum lycopersicum*) and facilitating feeding and promoting larval growth.^7^ Additionally, most insects cannot synthesize the enzymes required to degrade the polymers in plant cell walls, such as cellulases, pectinases, and ligninases.^8^ The gut microbiota compensates for these enzymatic deficiencies to aid digestion. For example, wood‐feeding wasps,^9^ leaf beetles,^10^ and wood‐feeding beetles^11^ rely on symbiotic microbes to produce plant cell wall degrading enzymes. These findings highlight the role of symbiotic microbes in facilitating insect adaptation and diversification by participating in specific metabolic pathways that overcome biochemical challenges posed by plant hosts.

The polysaccharide network constituting the plant cell wall represents the largest organic carbon reservoir on Earth, and pectin is one of the most complex polymers within this structure.^12^ Embedded within the cellulose and hemicellulose matrix, pectin is a major component of the middle lamella that connects cells, provides rigidity to the primary cell wall. Pectin plays a crucial role in maintaining the integrity, adhesion, and signal transduction of plant cells. It acts as a formidable barrier preventing parasites and pathogens from accessing the cytoplasm.^13^ During the digestion of plant cell walls, pectin must first be degraded to enable cellulases and hemicellulases to access their substrates, ultimately liberating plant cells from their protective walls. It is typically depolymerized by enzymes such as polygalacturonases (PGs), pectate lyases (PLs), and pectin methyl esterases (PMEs).^14^ Most pectinolytic enzymes have been annotated within microbial genomes and are generally classified as glycoside hydrolases, which can cleave glycosidic bonds in polysaccharides through single‐ or double‐displacement mechanisms.^15^, ^16^ For instance, γ‐proteobacteria found in the honeybee gut possess genes encoding pectinolytic enzymes, which are thought to facilitate the breakdown of pollen walls, significantly contributing to the genetic and functional diversification of honeybee‐specific bacteria.^17^ Furthermore, the symbiont *Stammera* provides pectinase for *Cassida rubiginosa* to contribute pectinolytic metabolism in plant‐derived diets, illustrating a striking example of symbiosis and evolutionary adaptation.^10^

*Zeugodacus cucurbitae*, classified under the order Diptera and family Tephritidae, was first identified in India in 1913.^18^ Subsequently, it has been documented in temperate, tropical, and subtropical regions of Asia, Africa, the Americas, and Australia.^19^, ^20^, ^21^ *Z. cucurbitae* is recognized as one of the most destructive pests of vegetables and fruits. It can affect up to 39 families and more than 130 plant species and its diet predominantly consists of plant fruits, especially cucurbit crops.^22^ The extent of losses varies between 30% to 100%, depending on the cucurbit species and the season.^19^ Its larvae had scratching mouthparts. When feeding, it first scrapes the food surface with oral hooks and then ingests sap and solid debris. A portion of food may undergo external digestion before ingestion. Pectin, a fundamental component of plant cell walls, serves as a critical substrate for enzymatic degradation during the digestion and utilization of host plants by *Z. cucurbitae*. Through genomic and transcriptomic analyses of 74 leaf beetle species and 50 symbionts, the results show that four fates for the ancestral‐type PG: maintenance, loss, replacement by horizontal gene transfer, and replacement by symbiont.^23^ However, whether the pectinolytic metabolism of *Z. cucurbitae* is mediated by symbiotic bacteria remains unexplored.

In the present study, we propose that the larval‐associated microbiota of *Z. cucurbitae* plays a critical regulatory role in larval growth and development by facilitating pectinolytic metabolism during feeding. To validate this hypothesis, we systematically characterized the microbial communities in larval feeding wounds and tissues through an integrated approach. 16S rRNA gene amplicon sequencing was performed to identify core symbiotic taxa. Functional isolation of culturable bacteria was conducted using LB and pectin medium. Bacterial reintroduction experiments were implemented to verify the correlation between pectinolytic bacteria and larval development. Our findings identified key symbiotic bacteria that participate in larval feeding processes *via* pectinase activity, thereby enabling efficient host plant utilization. This work not only elucidates microbiota‐mediated adaptation mechanisms underlying the ecological success of *Z. cucurbitae* on cucurbit hosts but also provides actionable targets for developing symbiont‐directed pest control strategies through disruption of pectinolytic metabolism pathways.

## 2 MATERIALS AND METHODS

### 2.1 Insects

Z*eugodacus cucurbitae* used in the experiments were continuously reared for more than 20 generations at the Plant Protection Research Institute of the Guangxi Academy of Agricultural Sciences. The insects were maintained in a rearing room under a photoperiod of 14/10 h light/dark, at 28 ± 2 °C and a relative humidity of 75% ± 5%. Larvae were fed on the host plant zucchini, *Cucurbita pepo* L. (purchased from Guangbai Supermarket). After emergence, adult flies were provided with a mixed diet (sucrose: yeast = 2:1) and reared in cages measuring 30 × 30 × 30 cm.

### 2.2 Effects of symbiotic bacteria on the growth and development of *Zeugodacus cucurbitae* larvae

Fresh zucchini with intact skin was sterilized in 75% ethanol for 3 min, followed by rinsing in sterile water for 1 min. Then, the zucchini was cut into blocks of 3 cm in height and placed in sterile tissue culture bottles for later use. The eggs were sterilized using 75% ethanol and 2.5% sodium hypochlorite, whereas the control group eggs were rinsed with sterile water for 3 min.^24^ The treated eggs were inoculated onto zucchini, with 30 eggs placed in each block. On the fifth day, the food consumption and the weight of mature larvae were measured. Larvae were collected with forceps and soaked in 75% alcohol overnight, then photographed with an Olympus stereoscope. Mature larvae were removed from the zucchini blocks and transferred to sterile fine sand in tissue culture bottles. The adult emergence initiated on the seventh day of the pupal stage. The pupal weight was measured on the third day of the pupal stage. Measurement of larval weight and pupal weight with an analytical balance. Each treatment was repeated thrice.

### 2.3 Collection of feeding wounds and tissue samples from larvae

The zucchini blocks were sterilized and placed in sterile Petri dishes. Then, they were exposed to 20‐day‐old adult fly cages for 30 min to collect the eggs. Eggs in the zucchini blocks were transferred to sterile tissue culture bottles for rearing. After 3 days of continuous larval feeding, approximately 100 mg of tissue from the feeding wounds of zucchini tissue (Zc_CpL) was collected using sterile tweezers, flash‐frozen in liquid nitrogen, and stored at −80 °C for later use. Then, the larvae were transferred to sterile Petri dishes and starved for 10 h. Subsequently, they were rinsed with 75% ethanol for 3 min, followed by sterile water for 1 min. Sterile 1× PBS was added to the Petri dishes, and the larvae were dissected under a microscope using sterile tweezers to obtain the head (Zc_Head), salivary gland (Zc_SG), foregut (Zc_FG), midgut (Zc_MG), and hindgut (Zc_HG) tissues. The tissues were collected from 30 larvae of each type. The resulting six samples were used for total DNA extraction as well as for the detection and isolation of culturable bacteria. Each sample consisted of six replicates.

### 2.4 Bacterial DNA extraction and 16S rDNA amplicon sequencing

Bacterial DNA from the feeding wound samples was extracted using the EasyPure Plant Genomic DNA Kit (Tansgen, Beijing, China) according to the manufacturer's instructions. DNA was extracted from each larval tissue sample using the TIANamp Genomic DNA Kit (Tiangen, Beijing, China). The extracted DNA was sent to Shanghai Paiseno Biotechnology Co. Ltd. for high‐throughput sequencing. The V3 + V4 regions of the bacterial 16S rRNA gene were amplified using NEB Q5 High‐Fidelity DNA Polymerase. The primers used were 338F (5′‐ACTCCTACGGGAGGCAGCA‐3′) and 806R (5′‐GGACTACHVGGGTWTCTAAT‐3′). The PCR amplification program included the following steps: an initial denaturation at 98 °C for 5 min, followed by 25 cycles of 98 °C for 30 s, 53 °C for 30 s, and 72 °C for 45 s, with a final extension at 72 °C for 5 min. The PCR products were subjected to paired‐end (PE250) sequencing on an Illumina NovaSeq sequencing platform.

### 2.5 Sequence analysis

Microbiome bioinformatics analysis was conducted using QIIME2 version 2019.4. Raw sequence data were demultiplexed using the demux plugin and primers were trimmed using the Cutadapt plugin. The DADA2 plug‐in was used for quality filtering, denoising, merging, and chimeric removal. The resulting sequences were clustered at 100% similarity to generate amplicon sequence variants (ASVs). Taxonomic classification of ASV feature sequences was performed by aligning them against reference sequences from the Greengenes database. Alpha diversity indices at the ASV level were calculated for each sample using QIIME software, whereas beta diversity was determined using the Jaccard distances and Bray‐Curtis distances to examine changes in the microbial community structure among the samples. The metabolic functions of the microbial communities were predicted using PICRUSt2 (Phylogenetic Investigation of Communities by Reconstruction of Unobserved States) based on the MetaCyc (https://metacyc.org/) and KEGG (https://www.kegg.jp/) databases (Gavin M. Douglas, *et al*., preprint).

### 2.6 Isolation and purification of cultivable bacteria

Feeding wounds and tissue samples from larvae were placed into 1.5 mL centrifuge tubes, with sample amounts matching those used for high‐throughput sequencing. Each sample was homogenized in 1 mL sterile 1× PBS. A 100 μL aliquot of the homogenate was added to 5 mL of liquid LB medium and incubated under enrichment conditions at 30 °C and 200 rpm for 12 h. From the bacterial suspension, 100 μL was serially diluted in 10‐fold gradients up to 10^−7^. Both undiluted and diluted suspensions (100 μL each) were spread onto LB agar plates and incubated aerobically at 30 °C for 12–24 h. All single colonies from plates with relatively uniform colony growth were selected and transferred to liquid LB medium for incubation at 30 °C and 200 rpm for 12 h. The selected colonies were further purified using the streak plate method, which was repeated twice to ensure purity. Purified bacterial cultures were mixed with an equal volume of 50% glycerol and stored at −80 °C for future use.

### 2.7 Screening and identification of pectinolytic bacteria

Primary screening of pectinolytic bacteria by Congo red staining.^25^, ^26^ The isolated single bacterial strains were cultured by inoculating 2 μL of each bacterial suspension onto pectin medium (composition: Pectin (Sigma‐Aldrich, Cat# P9135) 2.0 g, K₂HPO₄ 2.0 g, MgSO₄ 0.5 g, (NH₄)₂SO₄ 1.5 g, FeSO₄·7H₂O 0.01 g, ddH₂O 1000 mL, agar 20.0 g). The plates were incubated at 30 °C for 72 h, after which the colony diameters were measured. The plates were stained with 8 mg/mL Congo red for 30 min, followed by destaining with 1 M NaCl solution for 30 min. The bacteria forming clear zones were recorded, and the diameters of the clear zones were measured. Genomic DNA was extracted from bacterial suspensions using a TIANamp Bacteria DNA Kit (Tiangen, Beijing, China). PCR amplification of the bacterial 16S rRNA gene was performed using the universal primers 27F (5′‐AGAGTTTGATCCTGGCTCAG‐3′) and 1492R (5′‐TACGGTTACCTTGTTACGACTT‐3′). The amplified products were sequenced using sequencing services provided by Shanghai Sangon Biotech Co., Ltd. All bacterial strains isolated from feeding wounds were sequenced, whereas only strains with visible pectinase hydrolysis zones were sequenced for the other tissues. Nucleotide sequences were analyzed using the BLAST function in NCBI. Phylogenetic trees were constructed using MEGA 11 software and the neighbor‐joining method.

### 2.8 Determination of pectinase activity

Pectinase activity was determined by measuring the release of D‐galacturonic acid from pectin under assay conditions. The reducing groups were quantified by the 3,5‐dinitrosalicylic acid (DNS) method.^27^ Bacterial culture was precipitated by centrifugation at 12000 rpm at 4 °C for 15 min. The supernatant was used as crude enzyme for further studies. The reaction mixture contains 1 mL of 0.2% pectin (pectin dissolved in a K₂HPO₄‐KH₂PO₄ buffer solution, pH 6.5) and 0.5 mL of crude enzyme was incubated for 30 min at 46 °C. The mixture was mixed 1:1 with DNS reagent (Solarbio, Ca# D7800) and kept in a boiling water bath for 10 min. Then, rapid cooling in an ice bath is used to terminate the reaction. After centrifugation at 4 °C and 8000 *g* for 10 min, 200 μL of supernatant was transferred to a 96‐well microplate. Absorbance was read at 540 nm. Inactivated crude enzyme was used as the blank control, and each sample was measured in triplicate. Larval pectinase activity was determined as in this protocol. The larvae were ground with 1 mL of 1× PBS (10 larvae per treatment) and then centrifuged, and the supernatant was the crude enzyme. A standard curve was constructed using D‐galacturonic acid (Solarbio, Cat# G8120). One unit of enzyme activity (μ/mL) was defined as the amount of enzyme required to release 1 μg of D‐galacturonic acid per mL per hour under the assay conditions. The pectinase activity was calculated using the following formula^28^, ^29^:

$$\text{Pectinase Activities}\;\left(\mathrm{\mu g}/\mathrm{ml}/\text{hour}\right)=\frac{\text{Absorbance}\times G}{K\times \text{Time of incubation}\;\left(\text{hour}\right)}$$

Where *G* is the D‐galacturonic acid molecular weight, *K* is the slope of the standard curve for D‐galacturonic acid.

### 2.9 Bacterial reintroduction experiment

Quantitative analysis of the bacterial mixture was performed through optical density measurement and colony enumeration. The bacterial solution underwent serial dilution with 1× PBS at 1:1, 1:2, and 1:4 dilutions before determining the OD600. Subsequently, the diluted mixture was plated on solid medium and incubated for 12–16 h at 30 °C. Colony‐forming units (CFUs) were enumerated at the end of incubation. Based on the values of CFU and OD600, a simple linear regression was created. The required volume of sterile water for bacterial resuspension was calculated using the following formula^30^:

$$\begin{array}{l} \text{Sterile water volume for resuspension}\left(\mathrm{\mu L}\right)=\frac{\text{Absorbance of bacterial solution}\;\times \;\text{bacterial solution volume}\left(\mathrm{\mu L}\right)\;\times \;\text{Dilution factor}}{\text{Target}\;\mathrm{CFU}\;\text{concentration}} \end{array}$$

The bacterial mixture containing 10^8^ CFU was added to the sterile tissue culture bottle containing the zucchini block and 30 sterilized eggs. The bottles were incubated in a constant temperature incubator at 30 °C and 75% relative humidity. The weight of mature larvae was measured on day 5. The mature larvae were extracted from the zucchini and placed in sterile fine sand within the tissue culture bottles. Pupal weight was measured on day 3 after pupation. Each treatment was performed in triplicate.

### 2.10 Data statistics and analysis

Statistical analysis and visualization were performed using SAS 9.4 and GraphPad Prism 9.0 software. Differences between the two independent samples were analyzed using *T*‐tests. Multiple comparisons among samples were conducted using one‐way analysis of variance (ANOVA), followed by Duncan's multiple range test. Statistical significance was set at *P* < 0.05.

## 3 RESULTS

### 3.1 Effect of symbiotic bacteria removal on larval feeding and development

**Figure 1:**
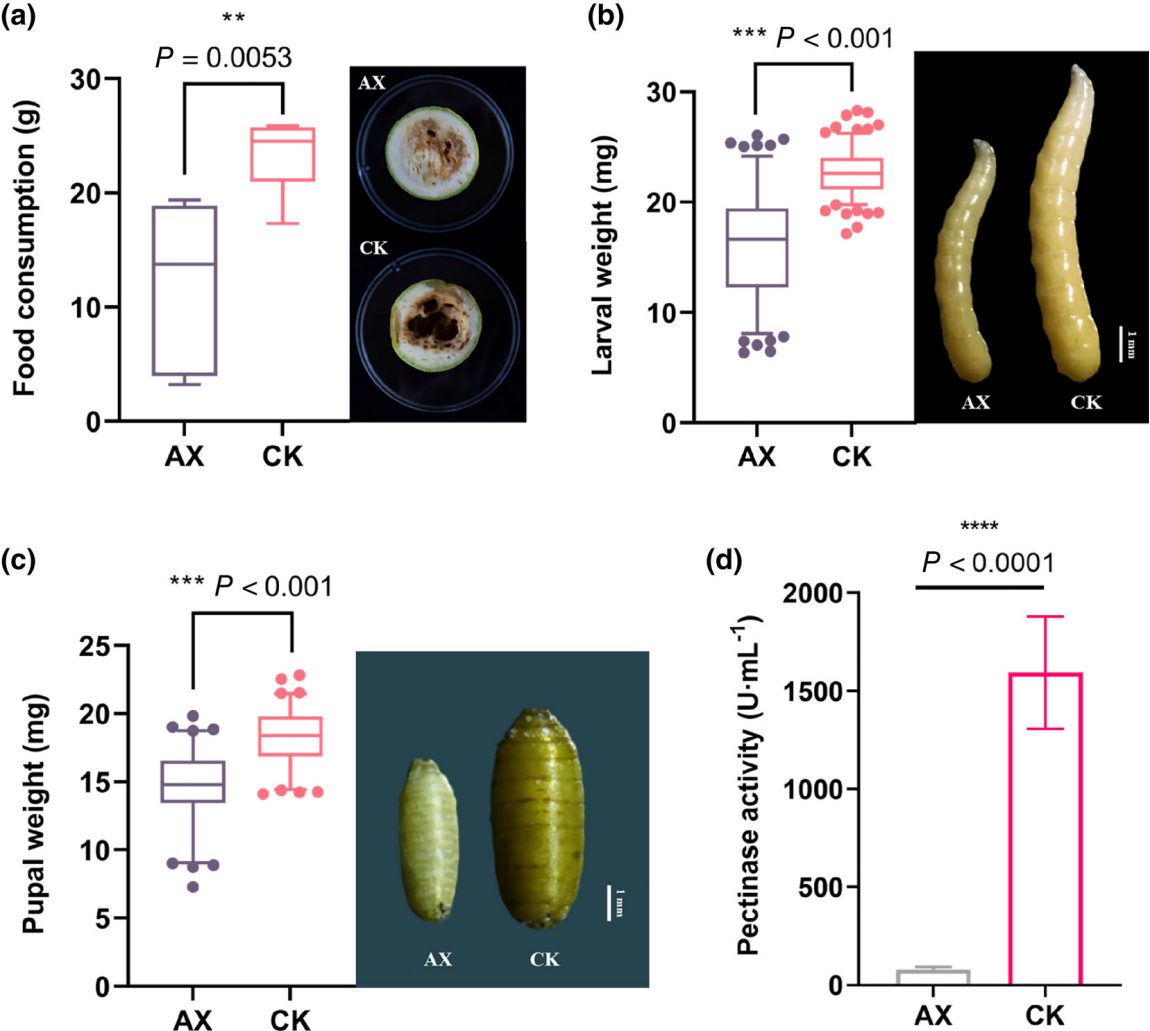
Effects of symbiotic bacteria removal on larvae. Changes in larval food consumption (a), mature larval weight (b), pupal weight (c), and pectinase activity (d) after the removal of symbiotic bacteria. Box and whisker plots (5–95 percentile). Points beyond the whiskers indicate potential extreme values. Histogram's values are presented as mean ± SD. Three replicates. Data were analyzed using an unpaired *T*‐test (**0.001 < *P* ≤ 0.01, **P ≤ 0.001).

After sterilizing the eggs (AX), the larvae were left to mature. Larvae were homogenized and plated on LB solid medium. The results show that culturable bacteria were successfully eliminated (Supporting Information, Fig. S1). Sterile eggs were transferred to fresh zucchini blocks. Compared to the conventional group (CK), the larvae from sterile eggs exhibited significantly reduced food consumption, and the degree of wound decay on the host plant was notably diminished (Fig. 1a). Additionally, the larval and pupal weights were significantly lower than those in the CK (Fig. 1b, c). Notably, the pectinase activity of AX larvae was significantly lower compared to the CK, with conventional larvae exhibiting up to 20 times higher pectinase activity (Fig. 1d). These findings indicate that the bacteria are involved in pectinolytic metabolism during the feeding process, benefiting *Z. cucurbitae* larval growth and development.

### 3.2 Bacterial community composition in the feeding wounds and different tissues of *Zeugodacus cucurbitae* larvae

**Figure 2:**
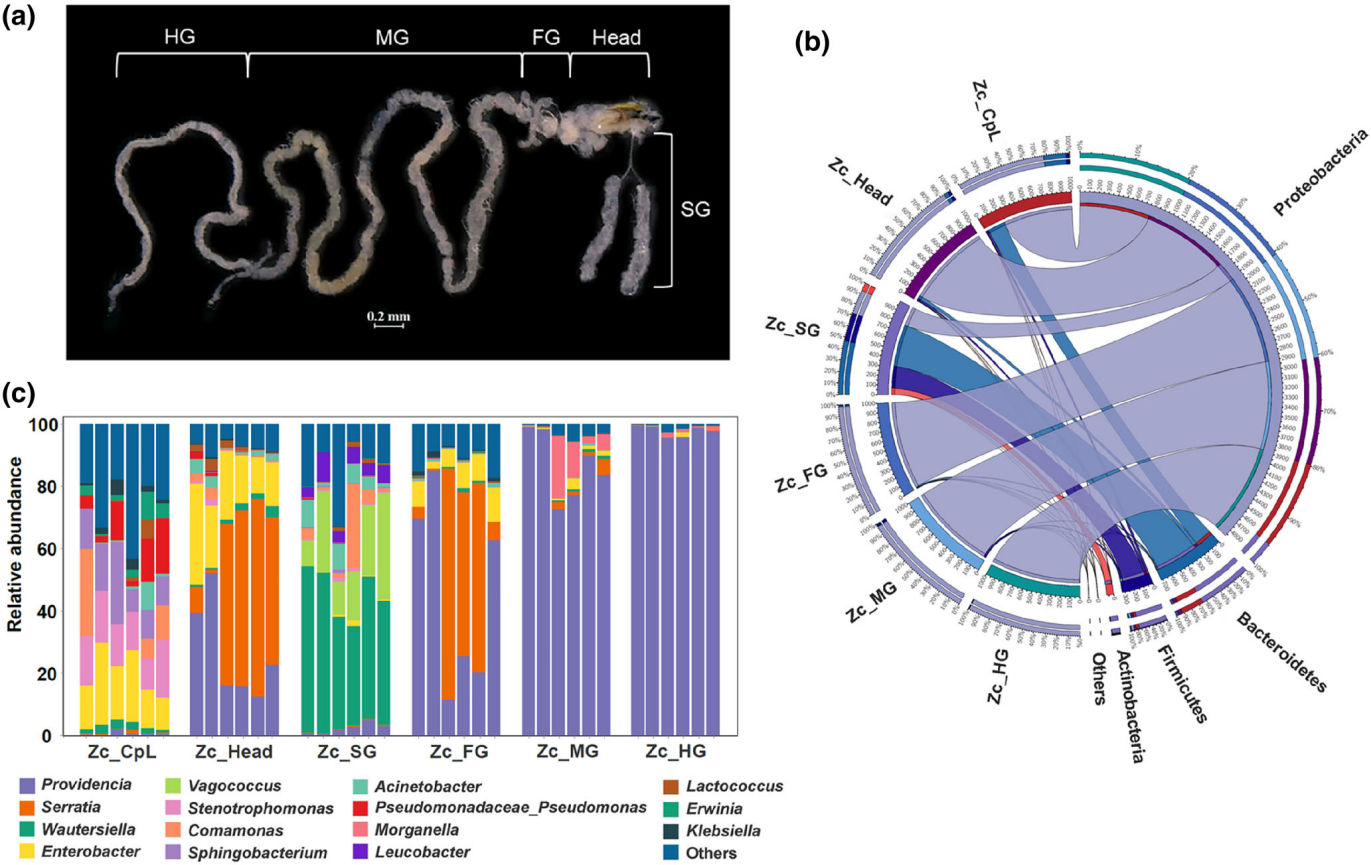
Analysis of microbial composition across the sample groups. (a) Sampling scheme for larval tissues of Z*eugodacus cucurbitae*. (b) Distribution of microbial communities at the phylum level across the samples, visualized using Circos. (c) Distribution of microbial communities at the genus level across the samples. Feeding wounds (Zc_CpL), larval heads (Zc_Head), salivary glands (Zc_SG), foregut (Zc_FG), midgut (Zc_MG), and hindgut (Zc_HG).

To investigate whether bacteria obtained from the feeding wounds were present within the larvae, six types of samples were collected: feeding wounds, larval heads, salivary glands, foreguts, midguts, and hindguts (Fig. 2a). The bacterial community composition in the feeding wounds and larval tissues was analyzed. Analysis of the sequencing results revealed that the rarefaction curves for all sample groups flattened as the sequencing depth increased, indicating that the sampling effort was sufficient and that the sequencing results reliably reflected the diversity of the samples (Supporting Information, Fig. S2).

Proteobacteria, Bacteroidetes, Firmicutes, and Actinobacteria were the predominant phyla across the different sample groups (Fig. 2b). Proteobacteria were the most dominant phylum in the feeding wounds, larval heads, foreguts, midguts, and hindguts. Bacteroidetes was the dominant phylum in the salivary glands and was identified as the second most dominant phylum in feeding wounds.

The bacterial genera *Providencia*, *Serratia*, *Wautersiella*, *Enterobacter*, and *Vagococcus* comprised the majority of microbial communities across the six groups. *Providencia* was the most abundant species (average relative abundance of 43.30%) followed by *Serratia* (average relative abundance of 12.26%). However, the dominant genera varied among the sample groups. *Enterobacter*, *Serratia*, *Wautersiella*, and *Providencia* were dominant in the feeding wounds, head, salivary glands, foregut, midgut, and hindgut, respectively (Fig. 2c).

**Figure 3:**
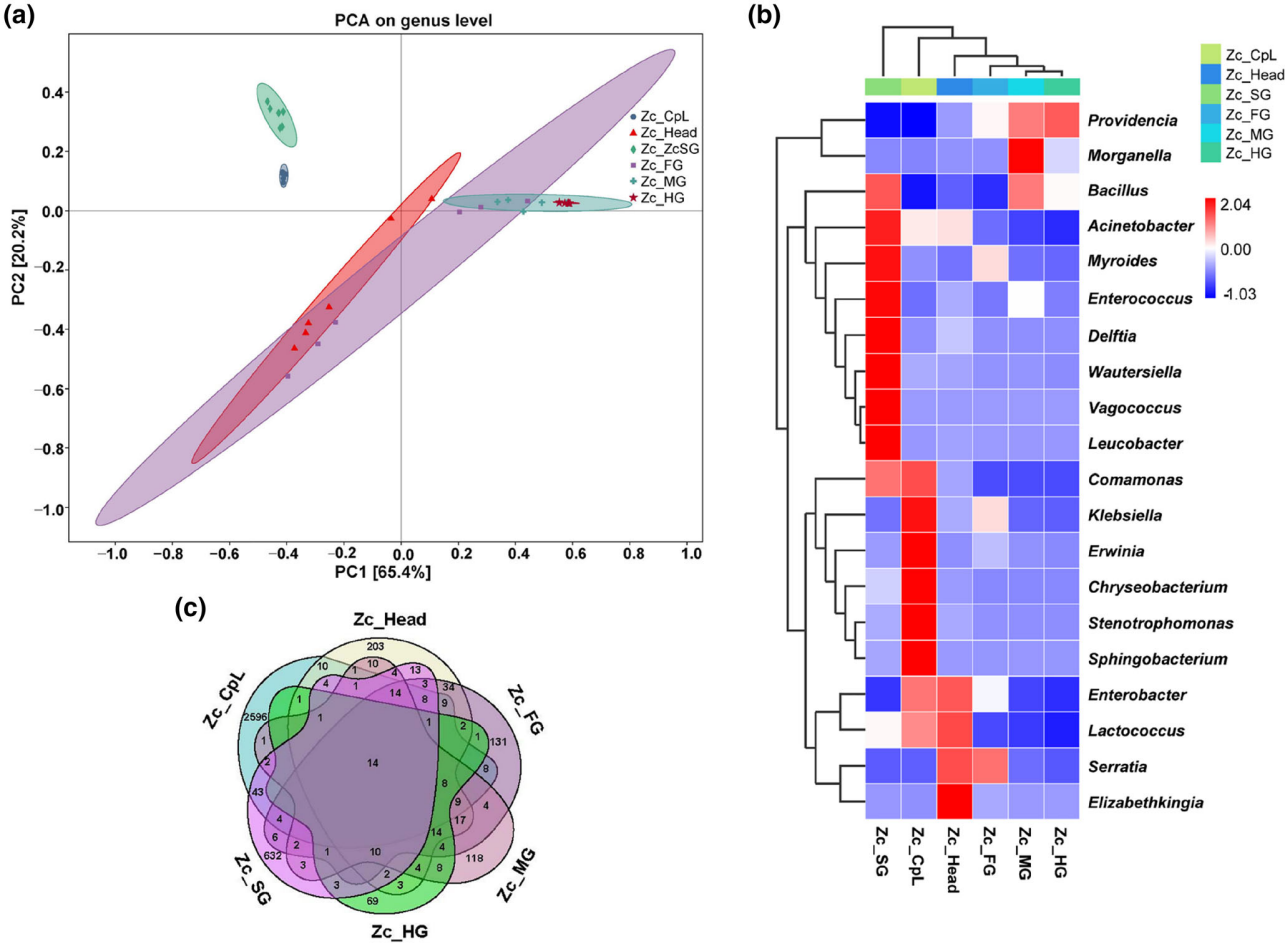
Comparison of microbial diversity across sample groups. (a) Principal component analysis (PCA) of microbial communities based on genus‐level data. (b) Top 20 genera in relative abundance across feeding wounds and larval tissues. (c) Petal diagram based on ASV data for each sample group.

To better understand the relationship between the bacterial community composition in feeding wounds and the larval tissues, principal component analysis (PCA) was conducted at the genus level. PCA grouped the bacterial communities into six groups (Fig. 3a). The first two principal components, PC1 and PC2, accounted for 65.4% and 20.2% of the total variance contribution, respectively. Biological duplicate samples were clustered together. The feeding wounds did not overlap with the samples from each tissue of the larvae, indicating that significant differences in microbial community composition were observed between the feeding wounds and larval tissues. There was overlap between the larval head, foregut, midgut, and hindgut, indicating that there was no difference in the microbial community composition between these four samples. Additionally, alpha diversity analysis revealed significant differences in bacterial richness and diversity between feeding wounds and the midgut and hindgut of the larvae (Supporting Information, Fig. S3). Beta diversity analysis showed that the microbial community composition of the feeding wounds differed significantly from the rest of the samples in the corresponding dimensions (Supporting Information, Fig. S4).

A heatmap analysis of the 20 most abundant genera. When comparing the gut bacteria in the six sample groups, it was shown that *Klebsiella*, *Erwinia*, *Chryseobacterium*, *Stenotrophomonas*, and *Sphingobacterium* were specifically enriched in feeding wounds (Fig. 3b). *Acinetobacter*, *Comamonas*, *Klebsiella*, *Enterobacter*, and *Lactococcus* were highly abundant in feeding wounds, but also exhibited considerable abundance in other larval tissues. The petal diagram indicated that feeding wounds had the highest number of unique ASVs, with overlaps observed among the six sample groups (Fig. 3c). These findings suggest that some bacteria present in feeding wounds are also distributed across larval tissues.

### 3.3 Isolation and identification of pectinolytic bacteria

**Figure 4:**
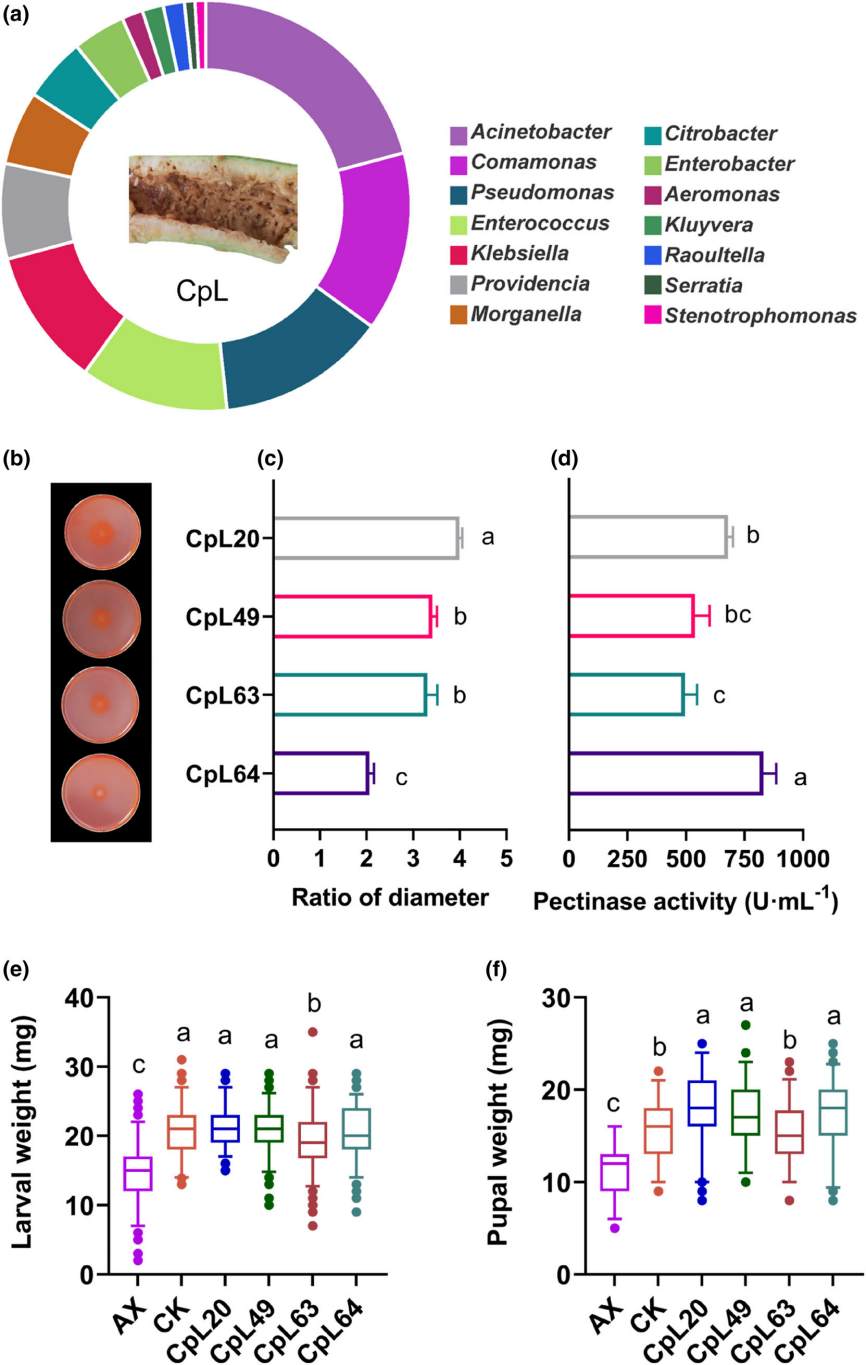
Isolation and characterization of pectinolytic metabolism bacteria. (a) Composition of culturable bacterial communities from feeding wounds. (b) Appearance of pectinase hydrolysis zones. (c) Ratio of hydrolysis zone diameter to colony diameter. (d) Pectinase activity. (e) Larval weight after inoculation with AX. (f) Pupal weight after inoculation with AX. In c–f, data were analyzed using one‐way ANOVA followed by Duncan's multiple range test. Histogram's values are presented as mean ± SD. Box and whisker plots (5–95 percentile). Points beyond the whiskers indicate potential extreme values. Three replicates. Lowercase letters above the bars and whiskers indicate statistically significant differences (*P* < 0.05).

A total of 120 culturable single bacterial strains were isolated and purified from the feeding wounds of zucchini consumed by *Z. cucurbitae* larvae. Additional strains were isolated from the larval tissues, with 70, 79, 81, 84, and 80 strains obtained from the head, salivary glands, foregut, midgut, and hindgut, respectively (Supporting Information, Fig. S5). Fourteen bacterial genera were isolated from the feeding wounds, and the five dominant genera were *Acinetobacter*, *Comamonas*, *Pseudomonas*, *Enterococcus*, and *Klebsiella* (Fig. 4a).

Among the isolated strains, four *Klebsiella* strains with pectinolytic metabolism abilities were identified in the feeding wounds (named CpL20, CpL49, CpL63, and CpL64). These four strains could grow on a medium with pectin as the sole carbon source and produced clear hydrolysis zones after Congo red staining (Fig. 4b). The diameters of the bacterial colonies and their hydrolysis zones ranged from 2.06 to 3.99 (Fig. 4c). The pectinase activities of the four strains ranged from 495.98 to 830.54 μ/mL (Fig. 4d), confirming their ability to utilize pectin. Bacterial strains with 16S rDNA sequences identical to those of CpL20 and CpL49 were isolated from the salivary glands, foregut, and hindgut, whereas CpL49 and CpL63 were isolated from the midgut. This demonstrated that certain bacteria identified in the feeding wounds were distributed across different tissues (Supporting Information, Table S1). Bacterial reintroduction experiments showed that four *Klebsiella* strains significantly promoted larval and pupal weight compared to the AX (Fig. 4e,f). Among them, strains CpL20, CpL49, and CpL64 exhibited relatively stronger growth‐promoting effects.

**Figure 5:**
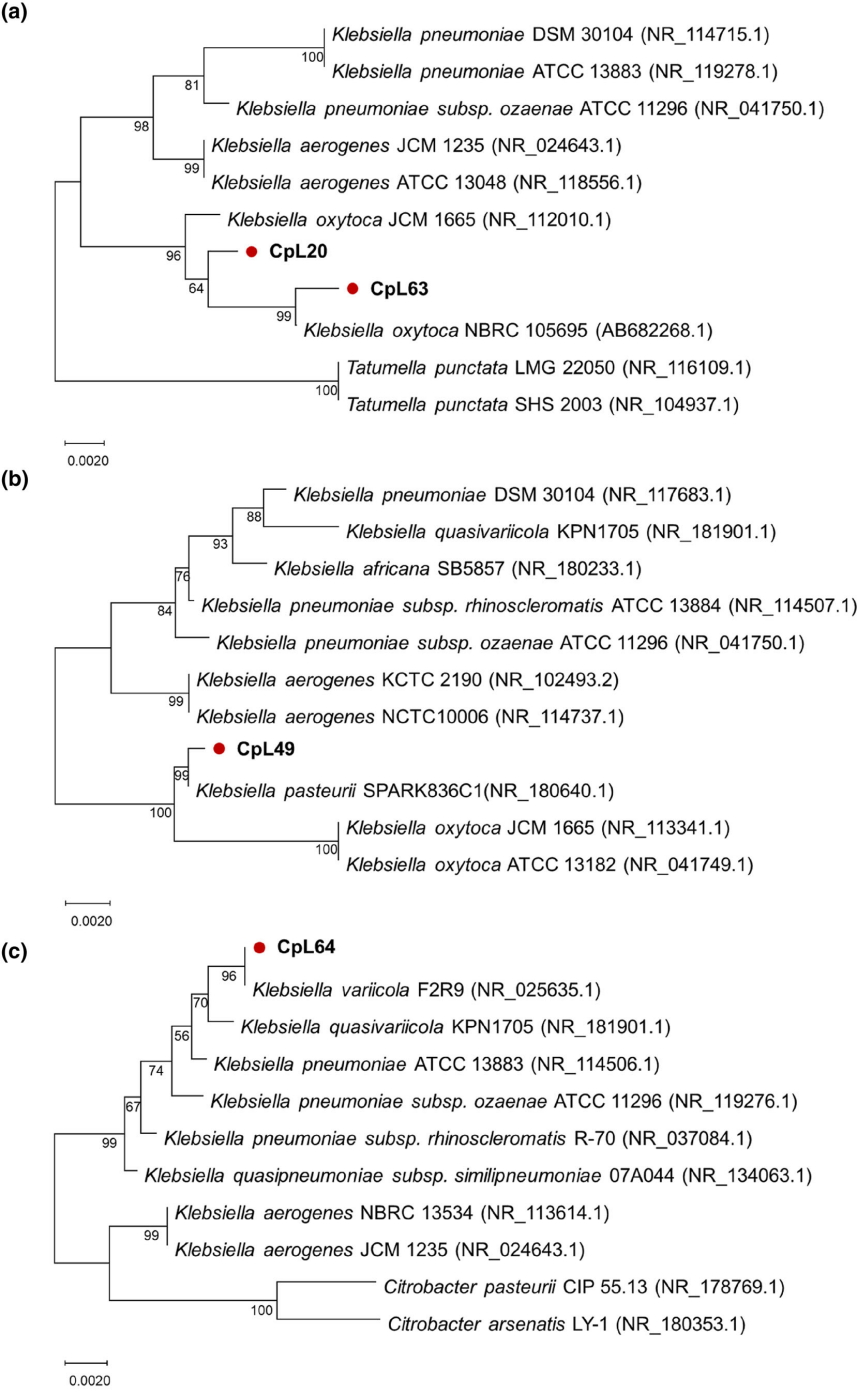
Phylogenetic trees based on 16S rRNA sequences. (a) Phylogenetic tree of CpL20 and CpL63. (b) Phylogenetic tree of CpL49. (c) Phylogenetic tree of CpL64. The phylogenetic trees were constructed using the neighbor‐joining method with 1000 bootstrap.

Phylogenetic analysis based on 16S rDNA sequences revealed CpL20, CpL49, CpL63, and CpL64 as *Klebsiella* species. The four strains' GenBank accession numbers were PV105608, PV105609, PV105610, and PV105611. The 16S rDNA sequence of the four strains showed 99.43%, 99.72%, 99.43%, and 100% similarity to *Klebsiella oxytoca* JCM 1665 (GenBank accession no. NR_112010.1), *K. oxytoca* NBRC 105695 (GenBank accession no. NR_180640.1), *Klebsiella pasteurii* SPARK836C1 (GenBank accession no. AB682268.1), and *Klebsiella variicola* F2R9 (GenBank accession no. NR_025635.1), respectively. The phylogenetic trees indicated that CpL20 and CpL63 were clustered with *K. oxytoca* (Fig. 5a), with CpL20 being genetically closer to *K. oxytoca* JCM 1665, whereas CpL63 was more closely related to *K. oxytoca* NBRC 105695. CpL49 was grouped with *K. pasteurii* SPARK836C1 (Fig. 5b), and CpL64 was clustered with *K. variicola* F2R9 (Fig. 5c). Preliminary identification results suggested that CpL20 and CpL63 represent two distinct strains of *K. oxytoca*, CpL49 is *K. pasteurii*, and CpL64 is *K. variicola*.

### 3.4 Potential functions of feeding wound microbiota and pectinases

**Figure 6:**
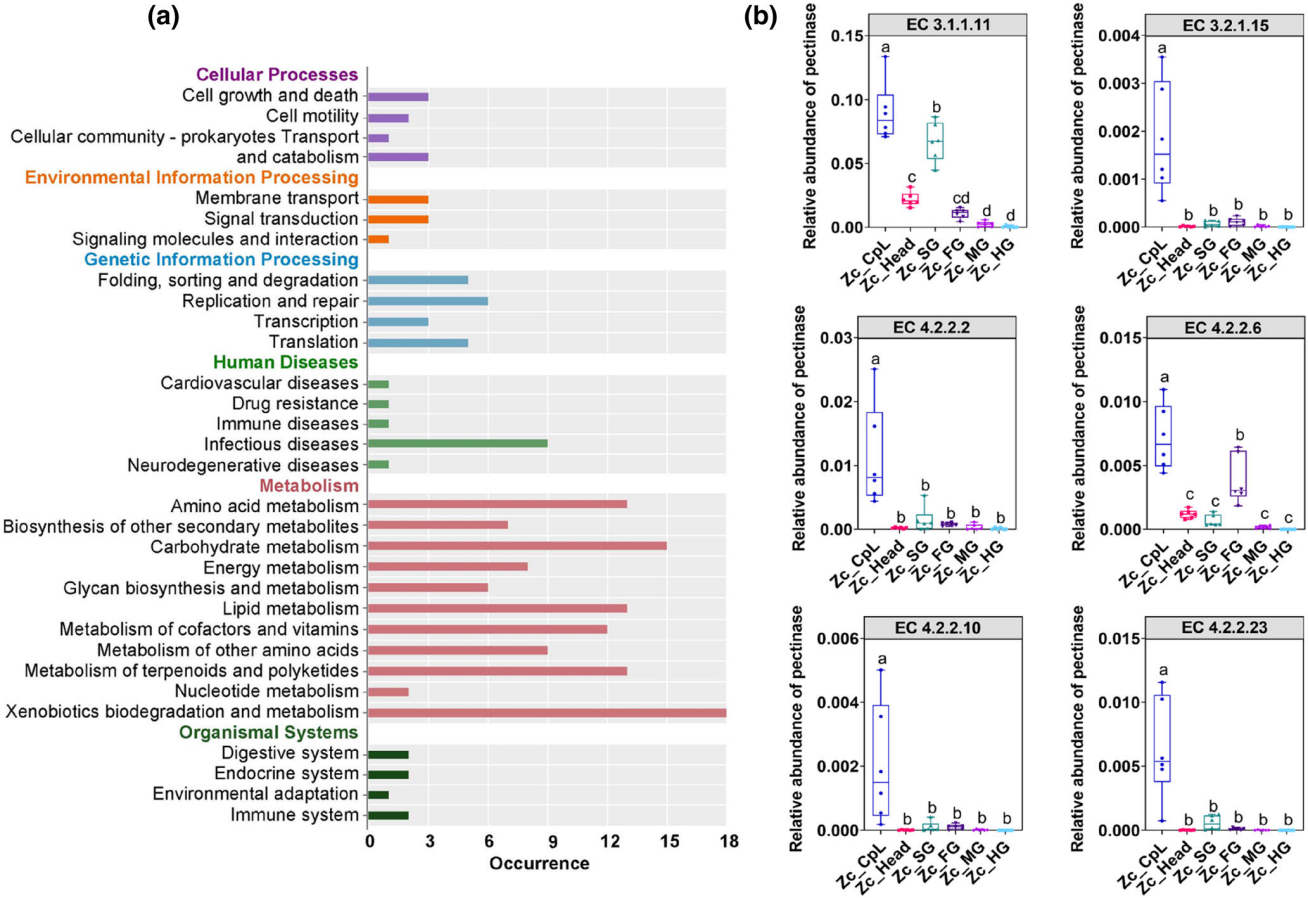
Potential functions and pectinase activity analysis of feeding wound‐derived microbiota. (a) Statistical analysis of metabolic pathways in the feeding wound microbiota based on the KEGG database. (b) Relative abundance of pectinases in feeding wounds and larval tissues. The types and abundances of various enzymes were predicted and analyzed using the MetaCyc database. The abundance values for each sample group are presented as ‘per million functional units’. Box and whisker plots (min to max). Display all data points. Data were analyzed using one‐way ANOVA followed by Duncan's multiple range test. Lowercase letters above the bars and whiskers indicate statistically significant differences (*P* < 0.05).

The potential functions of the microbial community were inferred based on the relative abundance of PICRUSt and were categorized into organismal systems, human diseases, cellular processes, environmental information processing, metabolism, and genetic information processing. Most of the pathways enriched in the microbial community of the feeding wounds of *Z. cucurbitae* larvae were involved in metabolism (Fig. 6a). Among the metabolic pathways, those related to carbohydrate metabolism were the second‐highest.

Microbial pectinases play a crucial role in pectinolytic metabolism during feeding. The relative abundances of pectinases were analyzed across the six sample groups (Fig. 6b). The results confirmed that the relative abundance of the six types of pectinases was the highest in the feeding wounds, followed by that in the salivary glands and foregut. Notably, EC 3.1.1.11 and EC 4.2.2.6 also showed a relatively high abundance in the head. Interestingly, the midgut and hindgut exhibited lower pectinase abundances, suggesting that the site of pectinolytic metabolism in the larval stage primarily depends on the anterior digestive system. This further suggests that symbiotic bacteria that provide pectinase to feeding wounds are more likely to originate from the salivary glands and foreguts of larvae.

## 4 DISCUSSION

Sterile *Z. cucurbitae* larvae exhibited reduced food consumption and a corresponding decrease in body weight, indicating that larval growth in natural environments depends on microbes. Symbiotic bacteria, with their extensive enzymatic and biosynthetic capabilities, assist the hosts in the digestion, assimilation, and metabolism of complex nutrients.^31^ Microbial communities supplement nutrition and promote insect growth. For example, the gut microbiota *Enterobacteriaceae cloacae via* the 4‐hydroxythreonine‐4‐phosphate dehydrogenase (*pdxA*) gene to synthesize vitamin B6 to promote *B. dorsalis* larval growth under nutrient‐deficient conditions.^30^ Symbiotic bacteria in reed beetles synthesize the most essential amino acids and B vitamins, such as riboflavin, during the larval stage, while pectinases encoded by symbionts support the leaf‐feeding capabilities of adult beetles.^32^ The symbiont *Stammera*, with its dynamic digestive enzyme repertoire, enables its host to digest a wide range of plant polysaccharides, thereby contributing to a broader ecological distribution.^33^ Symbiotic bacteria‐driven pectinolytic metabolism is essential for phytophagous insects. The pectinolytic ability was a determining factor in the evolution of leaf beetles as well as in weevils and longhorned beetles.^23^ Symbiont‐free beetle larvae exhibited significantly lower survivorship to adulthood.^10^ Therefore, the removal of symbiotic bacteria led to a decline in the growth metrics of *Z. cucurbitae* larvae due to the absence of certain enzymes essential for the digestion and metabolism of plant polysaccharides. This hypothesis was supported by the fact that the pectinase activity of sterile larvae was significantly lower compared to conventional larvae. Overall, symbiotic bacteria play an essential role in driving the pectinolytic metabolism in *Z. cucurbitae* larvae.

Our data showed that *Providencia* accounted for the highest proportion of the overall genus‐level averages across the six sample groups. Similarly, the symbiotic bacteria of *Z. cucurbitae* at different developmental stages were analyzed and found that *Providencia* and *Comamonas* were the dominant genera during the larval stage, and the dominant genus in females is *Klebsiella*.^34^ In *B. dorsalis*, the symbiont *K. oxytoca* assists in driving nitrogen waste recycling (NWR).^35^ In the gut of *Spodoptera frugiperda* larvae, *Klebsiella* EMBL‐1 depolymerizes polyvinyl chloride (PVC) and uses it as its sole energy source.^36^ However, the mutualistic relationship between *Klebsiella* and *Z. cucurbitae* has not yet been reported. The isolated *Klebsiella* strains were able to degrade pectin and promote the growth of sterile larvae, providing preliminary evidence of the involvement of *Klebsiella* in pectinolytic metabolism in *Z. cucurbitae*. High‐throughput sequencing and single‐strain isolation and identification revealed that, although *Klebsiella* was not the most abundant genus in feeding wounds, three strains were found in the larvae. This indicates that these bacteria are transferred to the host plant through feeding behaviors and even circulate between the feeding environment and the digestive system. A notable example of such insect‐microbe cycling is the relationship between *Drosophila melanogaster* and *Lactobacillus plantarum*. The larvae ingest *L. plantarum*, which is transported through the midgut with food, and some bacteria are excreted and subsequently re‐ingested by the larvae, maintaining the symbiotic relationship.^37^ This cyclic mechanism facilitates the host's adaptation to ecological niches. These findings indicate that *Klebsiella spp*. and *Z. cucurbitae* larvae promote each other's survival and fitness, forming a mutualistic symbiotic relationship. However, the molecular mechanisms underlying the maintenance and regulation of this relationship remain to be elucidated in future studies. Vertical transmission of insect‐associated microbes is an environmental adaptation strategy. *Enterobacteriaceae* are the dominant bacterial groups in adult *B. dorsalis*, including *Citrobacter*, *Klebsiella*, *Providencia*, and *Enterobacter*. These bacteria are known to be vertically transmitted through parental deposition on egg surfaces.^38^, ^39^, ^40^ These findings suggest that CpL64, found in feeding wounds but not isolated from other larval tissues, may have been transferred to the host plant along with the eggs.

The removal of symbiotic bacteria resulted in a reduction in larval food consumption, indicating that the larvae's ability to utilize host polysaccharides was compromised. This suggests a potential correlation between symbiotic bacteria and polysaccharide utilization. Just as symbiotic bacteria encoding pectinases allow tortoise leaf beetles to overcome a greater diversity of plant polysaccharides, corresponding to a wider ecological distribution.^33^ *C. pepo* is rich in polysaccharides, with carbohydrates accounting for 33.91 g per 100 g of peel and pulp,^41^, ^42^ making it a potential carbon source. Polysaccharides in host glycans must be broken down before they can be utilized as energy sources, and carbohydrate metabolism is the primary pathway for rapid pectinolytic metabolism.^17^ In this study, carbohydrate metabolism and transport were the primary metabolic pathways enriched in the microbial community of the feeding wounds. These findings suggest that carbohydrate metabolism is a key pathway by which the microbial community in feeding wounds contributes to pectinolytic metabolism.

Polygalacturonase is a major pectinase targeting nature's most abundant pectin class, homogalacturonan (HG).^33^ During pectinolytic metabolism, endo‐polygalacturonase (EC 3.2.1.15) and exo‐polygalacturonase (EC 3.2.1.67) hydrolyze nonesterified HG, whereas pectate lyase (EC 4.2.2.2) cleaves glycosidic bonds at the O‐4 position of galacturonate residues.^43^ Pectin methyl esterase (EC 3.1.1.11) removes methyl groups, whereas pectin lyase (EC 4.2.2.10) preferentially cleaves glycosidic bonds in methyl‐esterified galacturonate residues.^44^ In bacteria, oligogalacturonate lyase (EC 4.2.2.6) degrades oligogalacturonates in the cytoplasm.^45^ Additionally, rhamnogalacturonan lyase (EC 4.2.2.23) cleaves the bonds between the L‐rhamnosyl and α‐1,4‐galacturonosyl residues in RG‐I.^46^, ^47^ Using the MetaCyc database, all enzymes except exo‐polygalacturonase were identified in the feeding wounds. Pectinolytic enzymes secreted by bacteria in feeding wounds are essential for weakening plant cell walls and facilitating larval feeding. These findings highlight the significant role of symbiotic bacteria in the adaptation of *Z. cucurbitae* to plant‐based diets. The symbiont *Stammera* of *C. rubiginosa* degrades pectin using two enzymes, polygalacturonase (glycoside hydrolase family 28) and rhamnogalacturonan lyase (polysaccharide lyase family 4).^10^ Additionally, the magnesium/nickel/cobalt membrane transporter (CorA) is known to regulate pectinase production in λ‐proteobacteria and the general translocation membrane protein (SecA) with a demonstrated capacity for the extracellular export of pectinases in Gram‐negative bacteria.^10^, ^48^, ^49^ However, the specific mechanisms of pectinolytic metabolism by *Klebsiella spp*. require further investigation. In subsequent studies, successfully deciphering the symbiont‐mediated insect‐plant interactions in *Z. cucurbitae* could facilitate the targeted development of novel and highly effective insect control strategies. For instance, manipulating the insect's symbiotic microbiota could reduce the insect's adaptability to its host plants.

## 5 CONCLUSION

Our findings demonstrate that the microbes facilitate the growth and development of *Z. cucurbitae* larvae fed on host plants. Functional characterization of *Klebsiella spp*., including pectinase activity, spatial distribution patterns, and reintroduction experiments, revealed that larval‐derived symbiotic bacteria persistently colonize feeding wounds. These bacteria recycle between the larvae and the feeding wounds, actively participate in pectinolytic metabolism, and promote larval growth. Further studies are required to elucidate the underlying mechanisms. This study provides novel insights into the role of symbiotic bacteria in the growth of plant pests. It lays a foundation for exploring the relationship between *Z. cucurbitae* and its symbiotic bacteria, particularly the role of these bacteria in pectinolytic metabolism and their contribution to the ecological adaptation of phytophagous insects. Our work will provide a theoretical basis for the symbiont‐based control strategy of *Z. cucurbitae*.

## CONFLICT OF INTEREST

The authors declare no conflict of interest.

## Supporting information


**Data S1.** Supporting Information.

## Data Availability

The datasets presented in this study were deposited in the NCBI Sequence Read Archive under accession number PRJNA1224616.
