# Supplementary material for: Symbiotic bacteria participate in pectinolytic metabolism to enhance larval growth in Zeugodacus cucurbitae
Source: Pest Manag Sci. 2025 Jul 3;81(10):6820–31. doi: 10.1002/ps.70035 (PMC12441772; doi:10.1002/ps.70035)
Supplement: Supplementary file 1 — Data S1. Supporting Information. [file PS-81-6820-s001.docx]

**Supplementary Information**

**
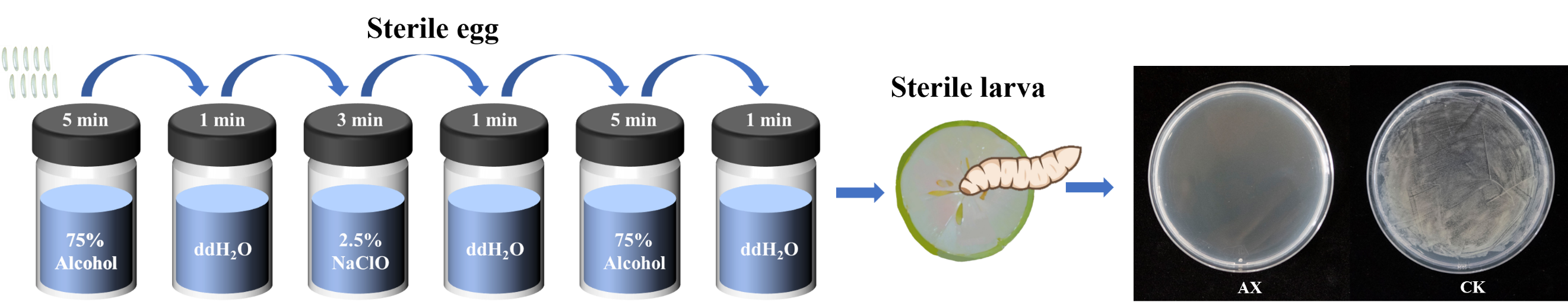
**

**Fig. S1 Procedure for the sterilization of eggs and the effectiveness of symbiotic bacteria removal in larvae**


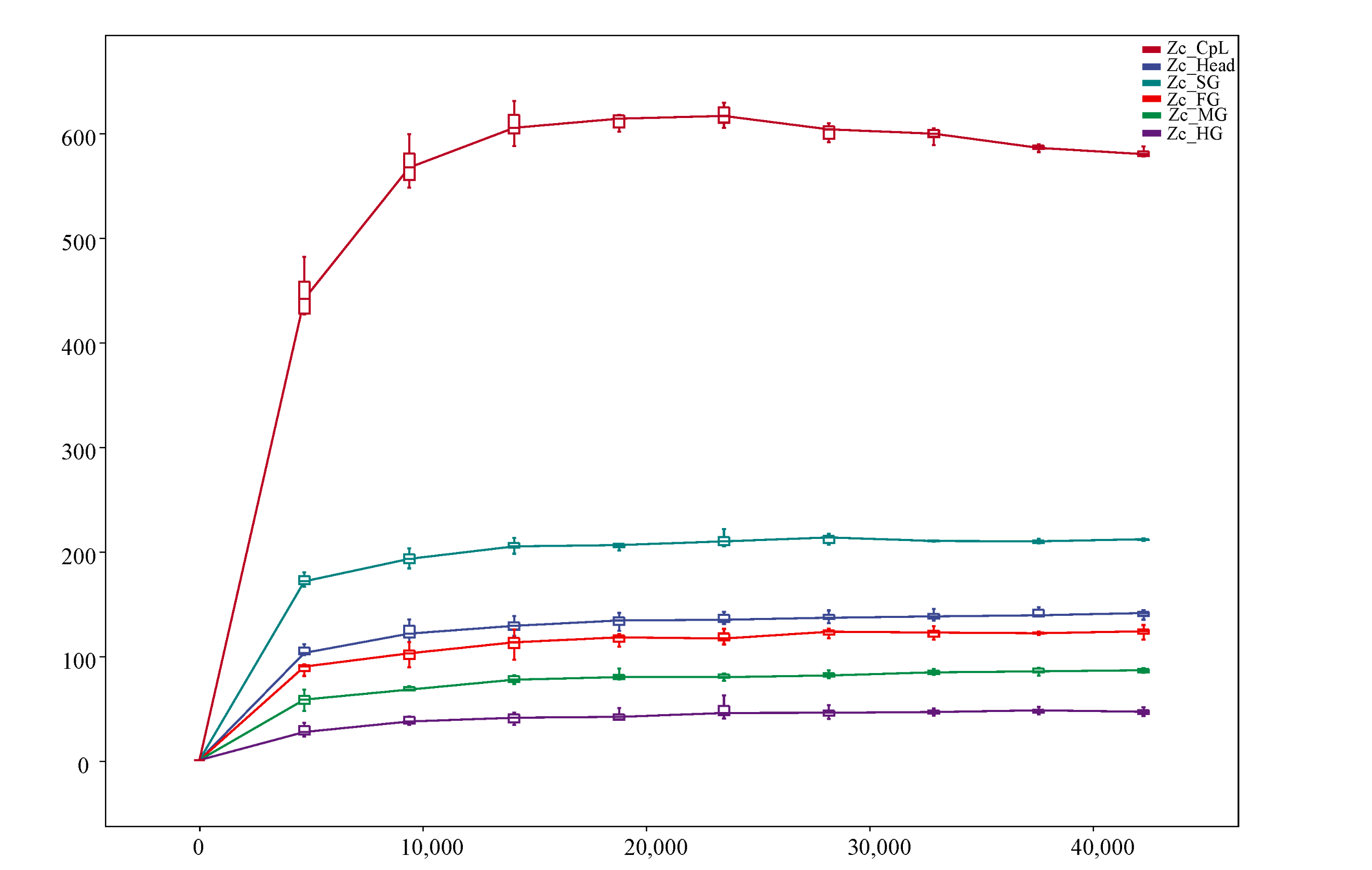


**Fig. S2 Rarefaction curve**

The x-axis displays the sequencing depth and the y-axis shows the median alpha diversity index calculated from 10 iterations, represented by box plots.

**
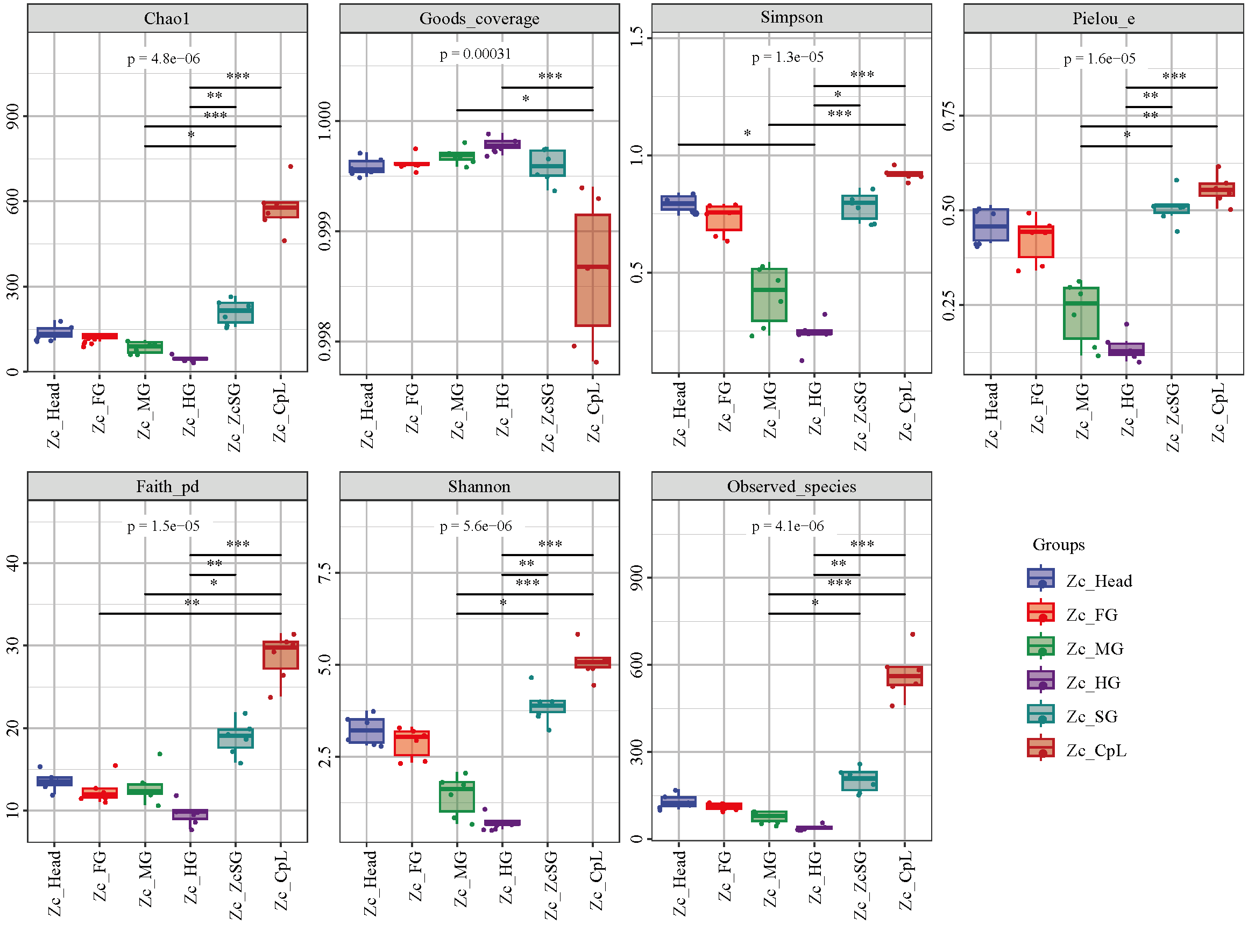
**

**Fig. S3 Microbial alpha diversity indices across sample groups**

Numbers below the diversity index labels represent the *p*-values obtained from the Kruskal-Wallis test results. The significance markers of the pairwise group comparisons conducted using Dunn’s post-hoc test are shown.


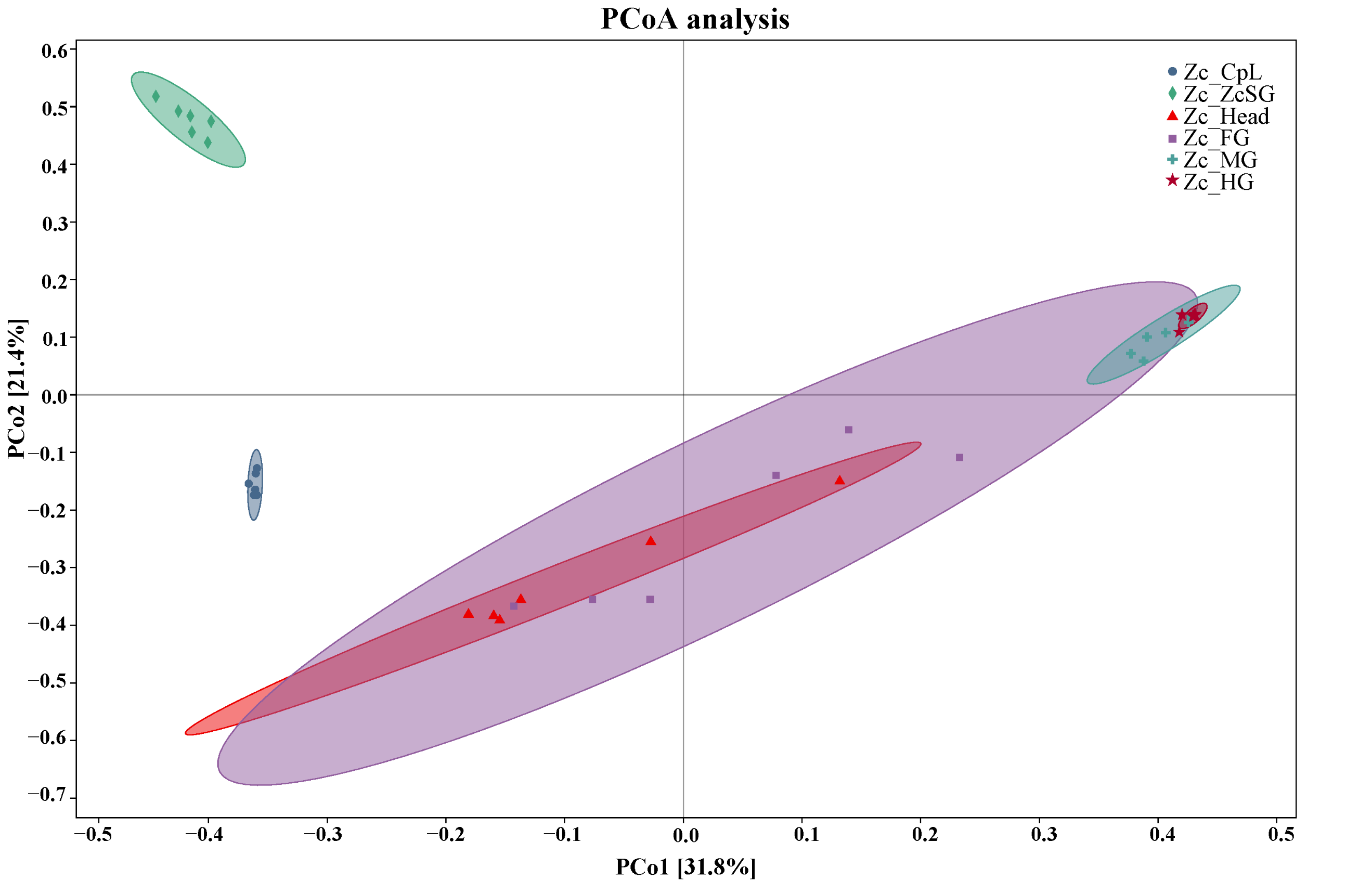


**Fig. S4 Distance matrix and principal coordinate analysis**

Using Jaccard and Bray-Curtis distances to asssess beta diversity.

**
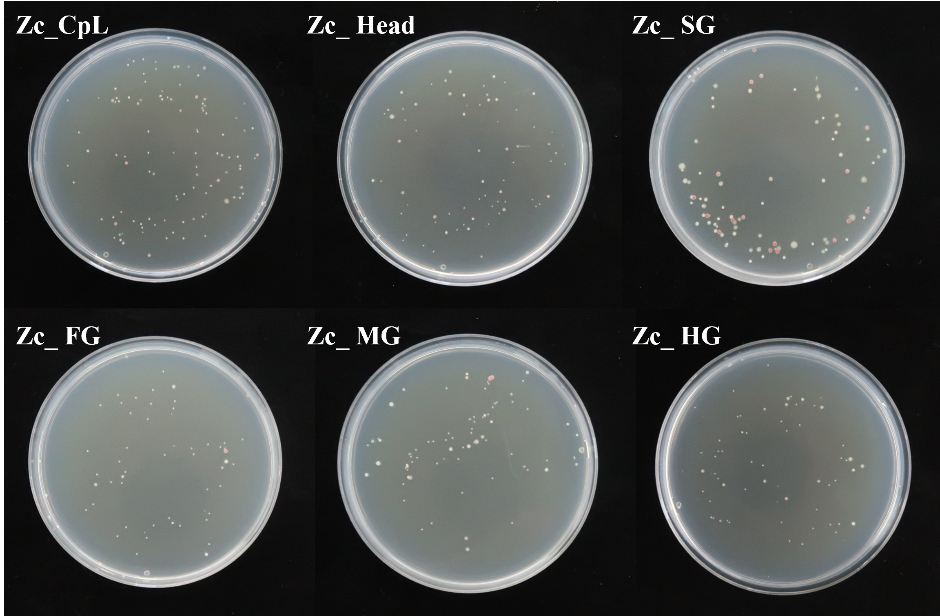
**

**Fig. S5. Colony distribution of the 10^-6 bacterial suspension on LB medium across samples**

**Table S1 Distribution of isolates across larval tissues**

| **Strain** | **Tissue** | | | | |
| --- | --- | --- | --- | --- | --- |
|  | Zc_Head | Zc_SG | Zc_FG | Zc_MG | Zc_HG |
| CpL20 | **-** | + | + | - | + |
| CpL49 | - | + | + | + | + |
| CpL63 | - | - | - | + | - |
| CpL64 | - | - | - | - | - |

“+” indicates presence, and “-” indicates absence.
